# Same-Day Versus Non-Simultaneous Extracorporeal Membrane Oxygenation Support for In-Hospital Cardiac Arrest Complicating Acute Myocardial Infarction

**DOI:** 10.3390/jcm9082613

**Published:** 2020-08-12

**Authors:** Saraschandra Vallabhajosyula, Sri Harsha Patlolla, Malcolm R. Bell, Wisit Cheungpasitporn, John M. Stulak, Gregory J. Schears, Gregory W. Barsness, David R. Holmes

**Affiliations:** 1 Department of Cardiovascular Medicine, Mayo Clinic, Rochester, MN 55905, USA; Bell.Malcolm@mayo.edu (M.R.B.); Barsness.Gregory@mayo.edu (G.W.B.); Holmes.David@mayo.edu (D.R.H.); 2 Division of Pulmonary and Critical Care Medicine, Department of Medicine, Mayo Clinic, Rochester, MN 55905, USA; 3 Center for Clinical and Translational Science, Mayo Clinic Graduate School of Biomedical Sciences, Rochester, MN 55905, USA; 4 Section of Interventional Cardiology, Division of Cardiovascular Medicine, Department of Medicine, Emory University School of Medicine, Atlanta, GA 30322, USA; 5 Department of Cardiovascular Surgery, Mayo Clinic, Rochester, MN 55905, USA; Patlolla.SriHarsha@mayo.edu (S.H.P.); stulak.john@mayo.edu (J.M.S.); 6 Division of Nephrology, Department of Medicine, University of Mississippi School of Medicine, Jackson, MS 55905, USA; wcheungpasitporn@gmail.com; 7 Division of Critical Care Anesthesiology, Department of Anesthesiology and Perioperative Medicine, Mayo Clinic, Rochester, MN 55905, USA; Schears.Gregory@mayo.edu

**Keywords:** acute myocardial infarction, extracorporeal membrane oxygenation, in-hospital cardiac arrest, eCPR

## Abstract

Background: Although extracorporeal membrane oxygenation (ECMO) is used for hemodynamic support for in-hospital cardiac arrest (IHCA) complicating acute myocardial infarction (AMI), there are limited data on the outcomes stratified by the timing of initiation of this strategy. Methods: Adult (>18 years) AMI admissions with IHCA were identified using the National Inpatient Sample (2000–2017) and the timing of ECMO with relation to IHCA was identified. Same-day vs. non-simultaneous ECMO support for IHCA were compared. Outcomes of interest included in-hospital mortality, temporal trends, hospitalization costs, and length of stay. Results: Of the 11.6 million AMI admissions, IHCA was noted in 1.5% with 914 (<0.01%) receiving ECMO support. The cohort receiving same-day ECMO (N = 795) was on average female, with lower comorbidity, higher rates of ST-segment-elevation AMI, shockable rhythm, and higher rates of complications. Compared to non-simultaneous ECMO, the same-day ECMO cohort had higher rates of coronary angiography (67.5% vs. 51.3%; *p* = 0.001) and comparable rates of percutaneous coronary intervention (58.9% vs. 63.9%; *p* = 0.32). The same-day ECMO cohort had higher in-hospital mortality (63.1% vs. 44.5%; adjusted odds ratio 3.98 (95% confidence interval 2.34–6.77); *p* < 0.001), shorter length of stay, and lower hospitalization costs. Older age, minority race, non-ST-segment elevation AMI, multiorgan failure, and complications independently predicted higher in-hospital mortality in IHCA complicating AMI. Conclusions: Same-day ECMO support for IHCA was associated with higher in-hospital mortality compared to those receiving non-simultaneous ECMO support. Though ECMO-assisted CPR is being increasingly used, careful candidate selection is key to improving outcomes in this population.

## 1. Introduction

In-hospital cardiac arrest (IHCA) is a rare but serious complication of acute myocardial infarction (AMI) [1,2]. Despite improvements in the care of AMI patients, those experiencing IHCA continue to have 50–60% in-hospital mortality [1,2]. In recent times, the use of extracorporeal membrane oxygenation (ECMO) for supporting cardiac arrest has increased to support refractory cardiac arrest and cardiogenic shock in this population [3,4,5]. ECMO is capable of supporting cardiac and respiratory function and provides 3–5 L of cardiac output support as well as dual ventricular support [6,7,8,9,10,11,12]. ECMO cannulation may be performed simultaneously with cardiopulmonary resuscitation (eCPR) or subsequently for ongoing ischemia or refractory cardiogenic shock [5]. Though eCPR has been evaluated for out-of-hospital cardiac arrest [13], there are limited data on the use of ECMO for IHCA [14]. Prior studies on IHCA are limited by small sample sizes and unclear timing of ECMO cannulation [14]. Though eCPR in IHCA is more feasible due to the proximity to catheterization laboratories, the availability of immediate defibrillation resources, and access to critical care nursing facilities, the outcomes with eCPR vs. delayed ECMO cannulation remain understudied. There are limited nationally representative data on the timing of and outcomes with ECMO support for IHCA complicating AMI. We sought to compare the outcomes of same-day ECMO insertion compared to non-simultaneous ECMO insertion for IHCA complicating AMI. We hypothesized that use of early same-day ECMO would be associated with improved outcomes due to greater CPR support (i.e., eCPR).

## 2. Material and Methods

The National (Nationwide) Inpatient Sample (NIS) is the largest all-payer database of hospital inpatient stays in the United States. NIS contains discharge data from a 20% stratified sample of community hospitals and is a part of the Healthcare Cost and Utilization Project (HCUP), sponsored by the Agency for Healthcare Research and Quality. Information regarding each discharge includes patient demographics, primary payer, hospital characteristics, principal diagnosis, up to 24 secondary diagnoses, and procedural diagnoses. These data are available to other authors via the HCUP-NIS database with the Agency for Healthcare Research and Quality.

During the period between 1 January 2000 and 31 December 2017, a retrospective cohort of admissions from the HCUP-NIS with a primary diagnosis of AMI (International Classification of Disease-9 Clinical Modification (ICD-9CM) 410.x; International Classification of Disease-10 Clinical Modification (ICD-10CM) I21.x-22.x) with concomitant IHCA (ICD-9CM 99.60, 99.63, ICD-10PCS 5A12012) was identified [1,2,6,9,15]. Prior studies have shown administrative codes to have a high sensitivity and specificity for identification of IHCA [1,2,15,16]. ECMO support was identified (ICD-9CM 39.65; ICD-10CM 5A15223) consistent with prior literature [6,9,10]. Deyo’s modification of the Charlson Comorbidity Index was used to identify comorbid diseases, and prior methodology was used to identify cardiac and non-cardiac procedures (Table S1) [1,2,6,9,15]. Similar to prior work from the HCUP-NIS, the timing of IHCA and ECMO were calculated using the day of the procedure, and simultaneous ECMO insertion was defined as that performed on the same hospital day as the IHCA [1,2,9,10,17,18].

The primary outcome of interest was in-hospital mortality. Secondary outcomes included temporal trends of in-hospital mortality, hospitalization costs, hospital length of stay, discharge disposition, and the use of durable left ventricular assist device or cardiac transplantation as an exit strategy.

### Statistical Analysis

As recommended by HCUP-NIS, survey procedures using discharge weights provided with the HCUP-NIS database were used to generate national estimates. Using the trend weights provided by the HCUP-NIS, samples from 2000–2011 were re-weighted to adjust for the 2012 HCUP-NIS re-design. Chi-square and *t*-tests were used to compare categorical and continuous variables, respectively. Univariable analysis for trends, predictors, and outcomes was performed, and results were represented as odds ratios (ORs) with 95% confidence intervals (CIs). Multivariable logistic regression analysis incorporating age, sex, race, primary payer, comorbidity, hospital region, multiorgan failure, cardiogenic shock, shockable rhythm, type of AMI, and cardiac and non-cardiac procedures was performed for in-hospital mortality. For the multivariable modeling, regression analysis with purposeful selection of statistically (liberal threshold of *p* < 0.20 in univariate analysis) and clinically relevant variables was conducted.

The inherent restrictions of the HCUP-NIS database related to research design, data interpretation, and data analysis were reviewed and addressed. Pertinent considerations include not assessing individual hospital-level volumes (due to changes to sampling design detailed above), treating each entry as an ‘admission’ as opposed to individual patients, restricting the study details to inpatient factors since the HCUP-NIS does not include outpatient data, and limiting administrative codes to those previously validated and used for similar studies. Two-tailed *p* < 0.05 was considered statistically significant. All statistical analyses were performed using SPSS v25.0 (IBM Corp, Armonk, NY, USA).

## 3. Results

**Figure 1:**
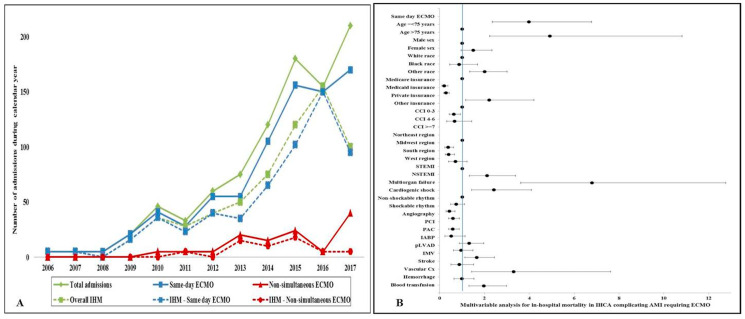
Extracorporeal membrane oxygenation (ECMO) use for acute myocardial infarction (AMI) complicated by in-hospital cardiac arrest. (**A**) Temporal trends of ECMO use and in-hospital mortality in the overall, same-day, and non-simultaneous ECMO placement; *p* < 0.001 for trend. (**B**) Multivariable in-hospital mortality for predictors of in-hospital mortality in AMI admissions with in-hospital cardiac arrest (IHCA).

During this 18-year period, there were 11,622,528 AMI admissions, of which IHCA was noted in 177,368 (1.5%). Of these IHCA admissions, ECMO support was used for 991 (<0.01%). The timing of IHCA and ECMO placement was available for 914 (92.2%) admissions, which constituted the final inclusion cohort. Same-day ECMO placement was performed in 795 (87.0%) admissions, and 119 (13.0%) received it non-simultaneously with their IHCA. In the non-simultaneous cohort, the median time to ECMO placement was 1 (interquartile range 1-1) hospital day after IHCA. The use of ECMO for IHCA started in 2005, with a rapid increase starting in 2008 (Figure 1A). Initially, the use of ECMO was limited to same-day support, with an increase in non-simultaneous cannulation starting in 2010 (Figure 1A). The baseline characteristics of those receiving simultaneous vs. non-simultaneous ECMO support for IHCA are presented in Table 1 and Table S2. The cohort receiving simultaneous ECMO was on average female, with lower comorbidity, higher rates of ST-segment elevation AMI, with a shockable rhythm, lower rates of multiorgan failure, and higher rates of mechanical and neurological complications (Table 1). Coronary angiography (67.5% vs. 51.3%; *p* = 0.001) was used more frequently, whereas concomitant mechanical circulatory support (intra-aortic balloon pump 42.1% vs. 61.3%, *p* < 0.001; percutaneous left ventricular assist device 20.8% vs. 31.1%, *p* = 0.02) was used less frequently in the same-day CPR group. The two groups had comparable rates of percutaneous coronary intervention (58.9% vs. 63.9%; *p* = 0.32) and pulmonary artery catheterization (6.2% vs. 4.2%; *p* = 0.53) use.

Compared to the cohort receiving non-simultaneous ECMO, the cohort receiving same-day ECMO had higher in-hospital mortality (63.1% vs. 44.5%; unadjusted OR 2.15 (95% CI 1.46–3.18); *p* < 0.001), shorter length of stay, lower hospitalization costs, and higher rates of palliative care consultation and do-not-resuscitate status use (Table 2). The temporal trends of in-hospital mortality in the overall, same-day, and non-simultaneous ECMO cohorts are presented in Figure 1A. Durable left ventricular assist device, cardiac transplantation, and discharge disposition were comparable between the two cohorts (Table 2). In a multivariable logistic regression analysis, same-day ECMO support was associated with higher in-hospital mortality (OR 3.98 (95% CI 2.34–6.77); *p* < 0.001) as compared to non-simultaneous ECMO cannulation (Figure 1B). Older age, minority race, non-ST-segment elevation AMI presentation, multiorgan failure, vascular complications, and need for blood transfusion predicted higher in-hospital mortality in IHCA complicating AMI (Figure 1B).

## 4. Discussion

In this large study looking at the use and outcomes of ECMO in IHCA complicating AMI, we noted <0.01% of all IHCA received ECMO support. The in-hospital mortality of AMI admissions receiving ECMO support for IHCA remained high, with worse outcomes in those receiving same-day support. Nearly 87% of ECMO placement was done on the same day. The cohort receiving same-day ECMO had higher use of palliative care and do-not-resuscitate status.

Use of same-day ECMO cannulation was associated with higher in-hospital mortality, contrary to our initial hypothesis. Though we cannot time the ECMO cannulation to the exact timing of CPR (i.e., confirm that this was indeed eCPR), this population appears to have higher morbidity, greater rates of care-limiting decisions, and shorter hospital length of stay. The lower rates of acute organ failure in this population may be explained by earlier in-hospital mortality, thereby preventing the evolution of the ‘hemo-metabolic’ cascade of shock and multiorgan failure [19]. It is conceivable that the use of same-day ECMO was a marker of worse comorbidity, higher acuity of illness, and a more refractory nature of the IHCA. Prior work has shown that the duration of the low-flow state was a significant predictor of higher in-hospital mortality in IHCA patients receiving VA-ECMO [14]. In addition to the in-hospital mortality, we note additional predictors of in-hospital in this population that are consistent with a prior systematic review and meta-analysis [14]. Our group and others have previously demonstrated shockable rhythms to be associated with lower in-hospital mortality. However, in this population receiving ECMO support, the type of rhythm did not influence in-hospital outcomes [1,2,15]. Furthermore, traditional risk factors such as older age, minority race, non-ST-segment elevation AMI, multiorgan failure, cardiogenic shock, and presence of complications were associated with higher in-hospital mortality. Lastly, the use of coronary angiography is consistent with the lower rates, as noted in high-risk patients from other studies [1,2].

### Limitations

This study has several limitations, some of which are inherent to the analysis of a large administrative database. The HCUP-NIS attempts to mitigate potential errors by using internal and external quality control measures. Information on the duration of the low-flow state, use of defibrillation and resuscitative medications, angiographic data, percutaneous coronary intervention location, revascularization failure, and time to CPR, which significantly influence outcomes, was not available in this database. There are limited data on patient- and family-specific limitations to therapeutic options, which may influence the clinical outcomes in this population in addition to the clinician’s judgment of patient prognosis. Despite these limitations, this study addresses significant knowledge on the contemporary use of ECMO for IHCA-complicating AMI.

## 5. Conclusions

ECMO is being increasingly used for the management of IHCA of AMI. Though the rates of use have increased, there has been limited incremental benefit in the outcomes of this vulnerable population. The use of ECMO in IHCA resuscitation has to be balanced against the risks, the complications, and resource utilization associated with this device [6,9,20].

## Figures and Tables

**Table 1:** Characteristics of same-day vs. non-simultaneous ECMO for IHCA in AMI.

Characteristic		Same-Day ECMO (N = 795)	Non-Simultaneous ECMO (N = 119)	*p*
Age (years)		62 (53–69)	63 (50–67)	0.43
Female sex		21.8	13.4	0.04
Race	White	56.2	60.5	<0.001
	Black	5.7	18.5	
	Others ^a^	38.2	21.0	
Charlson Comorbidity Index	0–3	56.0	58.0	0.10
	4–6	36.4	29.4	
	≥7	7.5	12.6	
AMI type	STEMI	70.6	68.1	0.59
	NSTEMI	29.4	31.9	
Rhythm	Shockable	70.2	83.2	0.003
	Non-shockable	29.8	16.8	
Cardiogenic shock		82.7	90.8	0.02
Acute non-cardiac organ failure	Multiorgan	87.2	95.0	0.01
	Respiratory	67.5	89.9	<0.001
	Renal	56.1	86.6	<0.001
	Hepatic	33.3	42.9	0.05
	Hematologic	43.2	39.5	0.49
	Neurologic	36.9	38.7	0.76
Complications	VSD	1.4	0.0	0.38
	PMR	0.6	0.0	>0.99
	Hemopericardium	1.4	0.0	0.38
	Cardiac tamponade	5.5	0.0	0.004
	Vascular	4.5	4.2	>0.99
	Limb amputation	0.0	5.0	<0.001
	Hemorrhagic	27.3	21.8	0.22
	Blood transfusion	34.3	17.6	<0.001
	Ischemic stroke	9.6	5.0	0.12
	ICH	2.5	0.0	0.10
Non-cardiac procedures	IMV	66.2	47.1	<0.001
	Acute hemodialysis	5.5	8.4	0.21
	Bronchoscopy	8.4	0.0	<0.001
	Electroencephalography	2.8	8.4	0.03

Represented as percentage or median (interquartile range); ^a^ Hispanic, Asian or Pacific Islander, Native American, Others. AMI: acute myocardial infarction; ECMO: extracorporeal membrane oxygenation; ICH: intracranial hemorrhage; IHCA: in-hospital cardiac arrest; IMV: invasive mechanical ventilation; NSTEMI: non-ST-segment-elevation myocardial infarction; PMR: papillary muscle rupture; STEMI: ST-segment-elevation myocardial infarction; VSD: ventricular septal defect.

**Table 2:** Clinical outcomes of same-day vs. non-simultaneous ECMO for IHCA in AMI.

Characteristic		Same-Day ECMO (N = 795)	Non-Simultaneous ECMO (N = 119)	*p*
In-hospital mortality		63.1	44.5	<0.001
Length of stay (days)		6 (2–14)	7 (2–26)	0.15
Hospitalization costs (×1000 USD)		341 (167–493)	482 (214–1000)	<0.001
Durable left ventricular assist device		4.5	4.2	>0.99
Cardiac transplantation		0.0	0.0	-
Palliative care consultation		16.6	0.0	<0.001
Do-not-resuscitate status		15.8	4.2	<0.001
Discharge disposition	Home	14.7	7.6	0.50
	Transfer	49.5	53.0	
	Skilled nursing facility	21.8	24.2	
	Home with HHC	14.0	15.2	
	Against medical advice	14.7	7.6	

Represented as percentage or median (interquartile range). AMI: acute myocardial infarction; ECMO: extracorporeal membrane oxygenation; IHCA: in-hospital cardiac arrest; HHC: home health care; USD: United States Dollars.

## Supplementary Materials

The following are available online at https://www.mdpi.com/2077-0383/9/8/2613/s1, Table S1: Administrative codes, Table S2: Additional baseline characteristics.

## Abbreviations

AMI	acute myocardial infarction
CI	confidence interval
CPR	cardiopulmonary resuscitation
ECMO	extracorporeal membrane oxygenation
eCPR	ECMO-assisted cardiopulmonary resuscitation
HCUP	Healthcare Cost and Utilization Project
ICD-9CM	International Classification of Diseases-9 Clinical Modification
ICD-10CM	International Classification of Diseases-10 Clinical Modification
IHCA	in-hospital cardiac arrest
NIS	National/Nationwide Inpatient Sample
OR	odds ratio

## References

[1] Vallabhajosyula S., Vallabhajosyula S., Burstein B., Ternus B.W., Sundaragiri P.R., White R.D., Barsness G.W., Jentzer J.C. (2020). Epidemiology of in-hospital cardiac arrest complicating non–ST-segment elevation myocardial infarction receiving early coronary angiography. Am. Heart J..

[2] Vallabhajosyula S., Vallabhajosyula S., Bell M.R., Prasad A., Singh M., White R.D., Jaffe A.S., Holmes D.R., Jentzer J.C. (2020). Early vs. delayed in-hospital cardiac arrest complicating ST-elevation myocardial infarction receiving primary percutaneous coronary intervention. Resuscitation.

[3] Bartos J.A., Carlson K., Carlson C., Raveendran G., John R., Aufderheide T.P., Yannopoulos D. (2018). Surviving refractory out-of-hospital ventricular fibrillation cardiac arrest: Critical care and extracorporeal membrane oxygenation management. Resuscitation.

[4] Chonde M., Sappington P., Kormos R., Althouse A.D., Boujoukos A. (2018). The Use of ECMO for the Treatment of Refractory Cardiac Arrest or Postarrest Cardiogenic Shock Following In-Hospital Cardiac Arrest: A 10-Year Experience. J. Intensive Care Med..

[5] Dennis M., Lal S., Forrest P., Nichol A., Lamhaut L., Totaro R.J., Burns B., Sandroni C. (2020). In-Depth Extracorporeal Cardiopulmonary Resuscitation in Adult Out-of-Hospital Cardiac Arrest. J. Am. Heart Assoc..

[6] Vallabhajosyula S., Bell M., Sandhu G.S., Jaffe A.S., Holmes J.D.R., Barsness G.W. (2020). Complications in Patients with Acute Myocardial Infarction Supported with Extracorporeal Membrane Oxygenation. J. Clin. Med..

[7] Vallabhajosyula S., Vallabhajosyula S., Vaidya V.R., Patlolla S.H., Desai V., Mulpuru S.K., Noseworthy P.A., Kapa S., Egbe A.C., Gersh B.J. (2020). Venoarterial Extracorporeal Membrane Oxygenation Support for Ventricular Tachycardia Ablation. ASAIO J..

[8] Vallabhajosyula S., O’Horo J.C., Antharam P., Ananthaneni S., Vallabhajosyula S., Stulak J.M., Dunlay S.M., Holmes D.R., Barsness G.W. (2020). Venoarterial Extracorporeal Membrane Oxygenation With Concomitant Impella Versus Venoarterial Extracorporeal Membrane Oxygenation for Cardiogenic Shock. ASAIO J..

[9] Vallabhajosyula S., Prasad A., Bell M.R., Sandhu G.S., Eleid M.F., Dunlay S.M., Schears G.J., Stulak J.M., Singh M., Gersh B.J. (2019). Extracorporeal Membrane Oxygenation Use in Acute Myocardial Infarction in the United States, 2000 to 2014. Circ. Heart Fail..

[10] Vallabhajosyula S., Prasad A., Sandhu G.S., Bell M.R., Gulati R., Eleid M.F., Best P.J.M., Gersh B.J., Singh M., Lerman A. (2019). Mechanical circulatory support-assisted early percutaneous coronary intervention in acute myocardial infarction with cardiogenic shock: 10-year national temporal trends, predictors and outcomes. EuroIntervention.

[11] Vallabhajosyula S., O Horo J.C., Antharam P., Ananthaneni S., Vallabhajosyula S., Stulak J.M., Eleid M., Dunlay S.M., Gersh B.J., Rihal C.S. (2018). Concomitant Intra-Aortic Balloon Pump Use in Cardiogenic Shock Requiring Veno-Arterial Extracorporeal Membrane Oxygenation. Circ. Cardiovasc. Interv..

[12] Vallabhajosyula S., Patlolla S.H., Sandhyavenu H., Vallabhajosyula S., Barsness G.W., Dunlay S.M., Greason K.L., Holmes D.R., Eleid M. (2018). Periprocedural Cardiopulmonary Bypass or Venoarterial Extracorporeal Membrane Oxygenation During Transcatheter Aortic Valve Replacement: A Systematic Review. J. Am. Heart Assoc..

[13] Yannopoulos D., Bartos J.A., Raveendran G., Conterato M., Frascone R.J., Trembley A., John R., Connett J., Benditt D.G., Lurie K.G. (2017). Coronary Artery Disease in Patients With Out-of-Hospital Refractory Ventricular Fibrillation Cardiac Arrest. J. Am. Coll. Cardiol..

[14] D’Arrigo S., Cacciola S., Dennis M., Jung C., Kagawa E., Antonelli M., Sandroni C.C. (2017). Predictors of favourable outcome after in-hospital cardiac arrest treated with extracorporeal cardiopulmonary resuscitation: A systematic review and meta-analysis. Resuscitation.

[15] Vallabhajosyula S., Jentzer J.C., Zack C.J. (2020). Cardiac Arrest Definition Using Administrative Codes and Outcomes in Acute Myocardial Infarction. Mayo Clin. Proc..

[16] Dezorzi C., Boyle B., Qazi A., Luthra K., Khera R., Chan P.S., Girotra S. (2019). Administrative Billing Codes for Identifying Patients With Cardiac Arrest. J. Am. Coll. Cardiol..

[17] Vallabhajosyula S., Patlolla S.H., Verghese D., Ya’Qoub L., Kumar V., Subramaniam A.V., Cheungpasitporn W., Sundaragiri P.R., Noseworthy P.A., Mulpuru S.K. (2020). Burden of Arrhythmias in Acute Myocardial Infarction Complicated by Cardiogenic Shock. Am. J. Cardiol..

[18] Vallabhajosyula S., Shankar A., Patlolla S.H., Prasad A., Bell M.R., Jentzer J.C., Arora S., Vallabhajosyula S., Gersh B.J., Jaffe A.S. (2020). Pulmonary artery catheter use in acute myocardial infarction-cardiogenic shock. ESC Heart Fail..

[19] Vallabhajosyula S., Dunlay S.M., Prasad A., Kashani K., Sakhuja A., Gersh B.J., Jaffe A.S., Holmes D.R., Barsness G.W. (2019). Acute Noncardiac Organ Failure in Acute Myocardial Infarction With Cardiogenic Shock. J. Am. Coll. Cardiol..

[20] Subramaniam A.V., Barsness G.W., Vallabhajosyula S., Vallabhajosyula S. (2019). Complications of Temporary Percutaneous Mechanical Circulatory Support for Cardiogenic Shock: An Appraisal of Contemporary Literature. Cardiol. Ther..

