# Supplementary material for: Same-Day Versus Non-Simultaneous Extracorporeal Membrane Oxygenation Support for In-Hospital Cardiac Arrest Complicating Acute Myocardial Infarction
Source: J Clin Med. 2020 Aug 12;9(8):2613. doi: 10.3390/jcm9082613 (PMC7465527; doi:10.3390/jcm9082613)
Supplement: Supplementary file 1 [file jcm-09-02613-s001.pdf]

Table S1. Administrative codes.

Comorbidity	International Classification of Diseases, 9.0 Clinical Modifications codes
Cardiac arrest	427.5
Cardiogenic shock	785.51
Respiratory failure	518.81, 518.82, 518.85, 786.09, 799.1, 96.7, 96.70, 96.71, 96.72
Hepatic failure	570.0, 572.2, 573.3, 573.4
Coronary angiography	37.22, 37.23, 88.53-88.56
Percutaneous coronary intervention	00.66, 36.01, 36.02, 36.05, 36.06, 36.07, 88.57
Invasive mechanical ventilation	96.7, 96.70, 96.71, 96.72
Hemodialysis	39.95
Vascular complications	904.0, 904.1, 904.2, 904.4, 904.40, 904.41, 904.7, 904.8, 904.9, 998.2, 999.2, 997.2, 997.7, 997.79, 447.0, 39.31, 39.41, 39.49, 39.52, 39.53, 39.56, 39.57, 39.58, 39.59, 39.79
Lower limb amputation	84.1, 84.10, 84.11, 84.12, 84.13, 84.14, 84.15, 84.16, 84.17, 84.18, 84.19
Post-operative hemorrhage/hematoma	998.11, 998.12, 285.1
Acute ischemic stroke	433.01, 433.11, 433.21, 433.31, 433.81, 433.91, 436.0, 437.1, 434, 434.0, 434.00, 434.01, 434.1, 434.10, 434.11, 434.9, 434.90, 434.91, 435, 435.0, 435.1, 435.2, 435.3, 435.8, 435.9
Intracranial hemorrhage	430, 431, 432.0, 432.1, 432.9
Acute kidney injury	584, 584.5, 584.6, 584.7, 584.8, 584.9

Table S2. Additional baseline characteristics.

Characteristic		Same-day ECMO (N=795)	Non-simultaneous ECMO (N=119)	*P*
**Primary payer**	**Medicare**	36.7	36.1	0.02
	**Medicaid**	13.0	4.2	
	**Private**	38.5	42.0	
	**Others[a]**	11.8	17.6	
**Quartile of median household income for zip code**	**0-25th**	18.0	26.1	0.01
	**26th-50th**	28.9	16.8	
	**51st-75th**	19.2	25.2	
	**75th-100th**	33.9	31.9	
**Prior coronary artery bypass grafting**		3.1	4.2	0.58
**Prior pacemaker**		0.0	4.2	<0.001
**Prior implantable cardioverter defibrillator**		1.9	5.0	0.05
**Weekend admission**		26.7	35.3	0.03
**Hospital teaching status and location**	**Rural**	1.4	0.0	0.04
	**Urban non-teaching**	3.9	8.4	
	**Urban teaching**	94.7	91.6	
**Hospital bed-size**	**Small**	8.6	4.2	0.21
	**Medium**	17.6	21.0	
	**Large**	73.8	74.8	
**Hospital region**	**Northeast**	37.7	36.1	0.16
	**Midwest**	20.5	13.4	
	**South**	25.8	33.6	
	**West**	16.0	16.8	

Legend: Represented as percentage or median (interquartile range); [a]Self-Pay, No Charge, Others.
Abbreviations: ECMO: extracorporeal membrane oxygenation.
